# Supplementary material for: Evaluation of the Anticancer Activity and Mechanism Studies of Glycyrrhetic Acid Derivatives toward HeLa Cells
Source: Molecules. 2023 Apr 2;28(7):3164. doi: 10.3390/molecules28073164 (PMC10095686; doi:10.3390/molecules28073164)

Evaluation of the Anticancer Activity and Mechanism Studies of Glycyrrhetic Acid Derivatives toward HeLa Cells

Ju Chen [1,†], Yunran Xu [1,†], Yan Yang [2,*], Xin Yao [1], Yuan Fu [1], Yi Wang [1], Yunjun Liu [1,3] and Xiuzhen Wang *

[1] *School of Pharmacy, Guangdong Pharmaceutical University, Guangzhou 510006, China; lyjche@gdpu.edu.cn (Y.L.)*

[2] *Department of Pharmacy, Guangdong Second Provincial General Hospital, Guangzhou 510317, China*

[3] *Guangdong Provincial Key Laboratory of Advanced Drug Delivery, Guangdong Provincial Engineering Center of Topical Precise Drug Delivery System, Guangdong Pharmaceutical University, Guangzhou 510006, China*

* Correspondence: yany@gd2h.org.cn (Y.Y.); wxzqq1234@gdpu.edu.cn (X.W.)

† These authors contribute equally to this work.

Supporting information

Figure S1 ^{1}H NMR spectra for **3a**

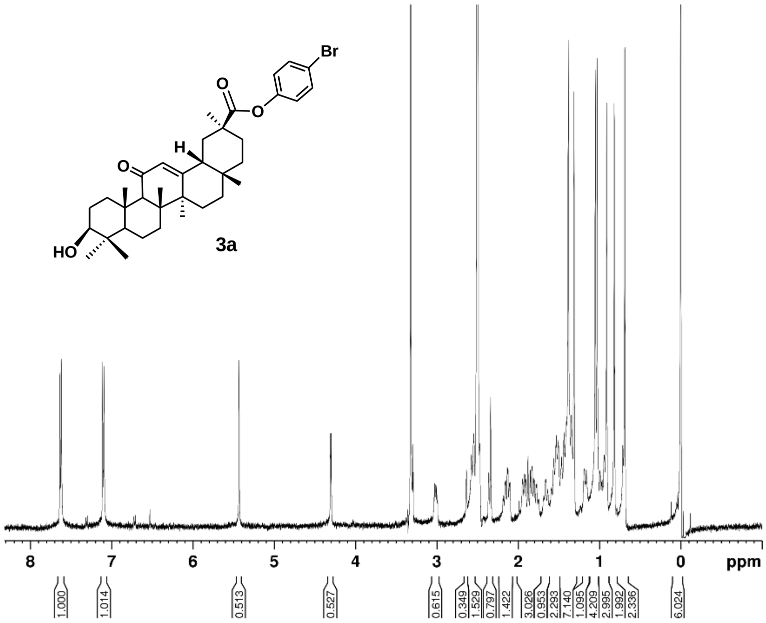

Figure S2 ^{13}C NMR spectra for **3a**

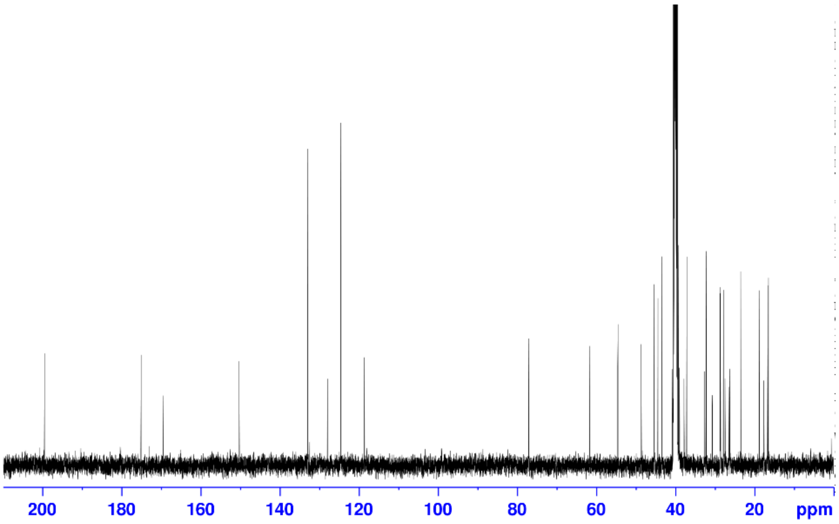

Figure S3 HRMS spectra for **3a**

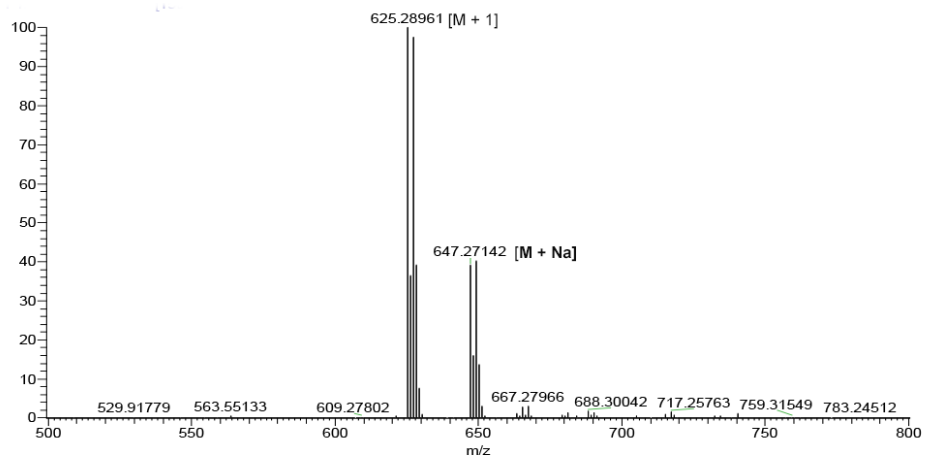

Figure S4 ^{1}H NMR spectra for **3b**

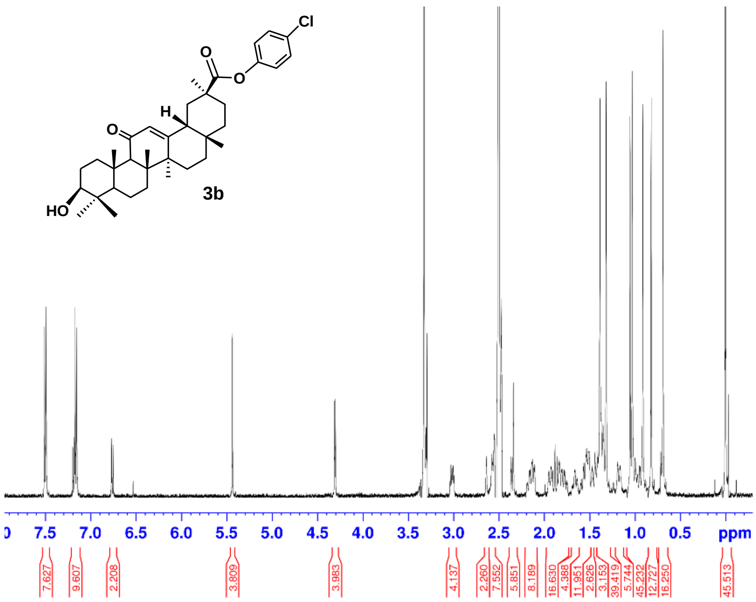

Figure S5 ^{13}C NMR spectra for **3b**

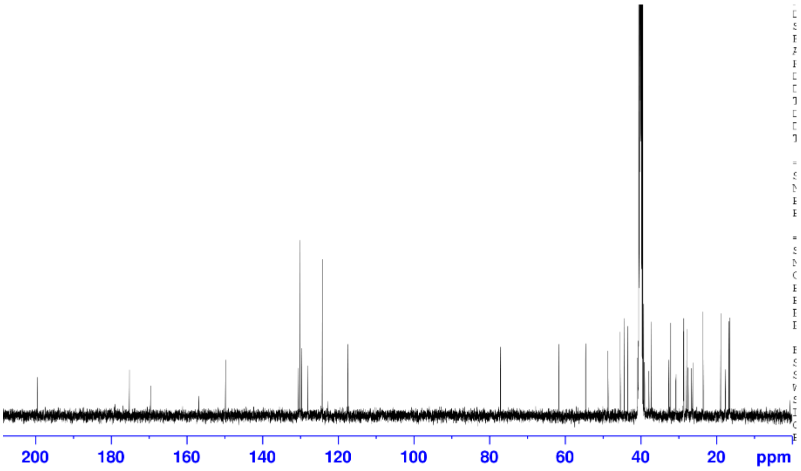

Figure S6 HRMS spectra for **3b**

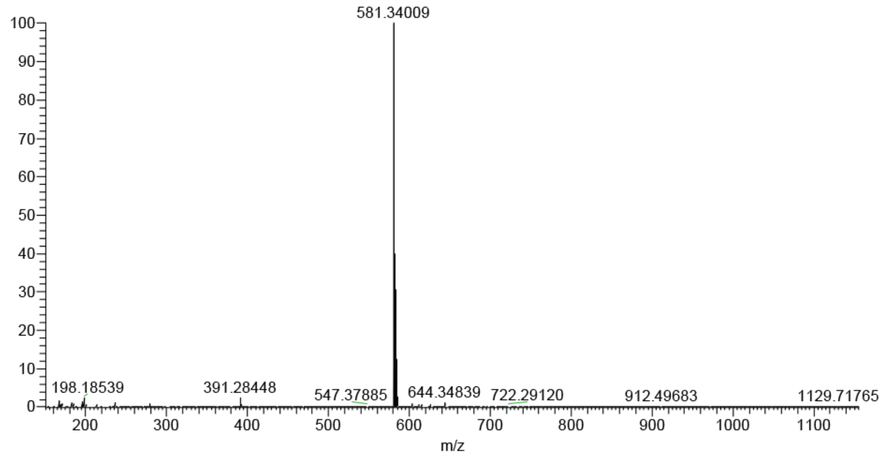

Figure S7 ^{1}H NMR spectra for **3c**

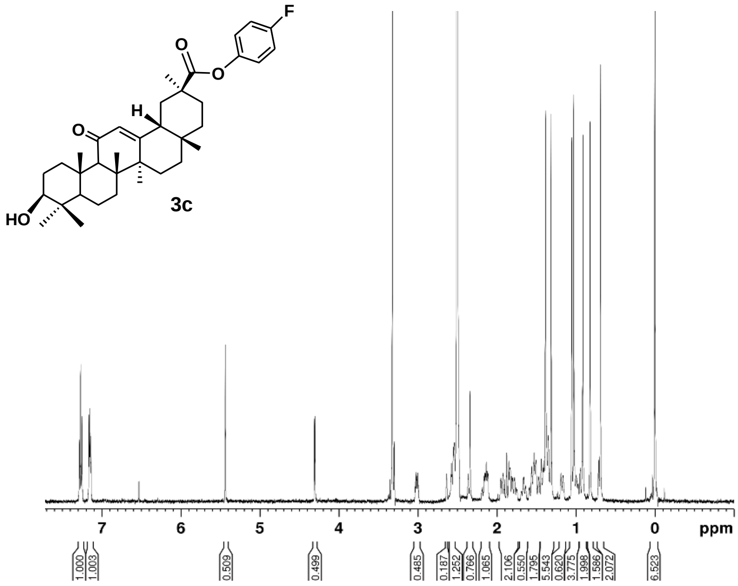

Figure S8 ^{13}C NMR spectra for **3c**

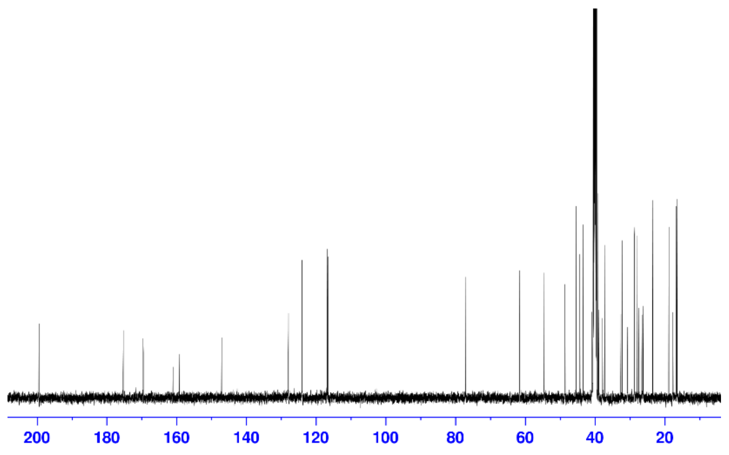

Figure S9 HRMS spectra for **3c**

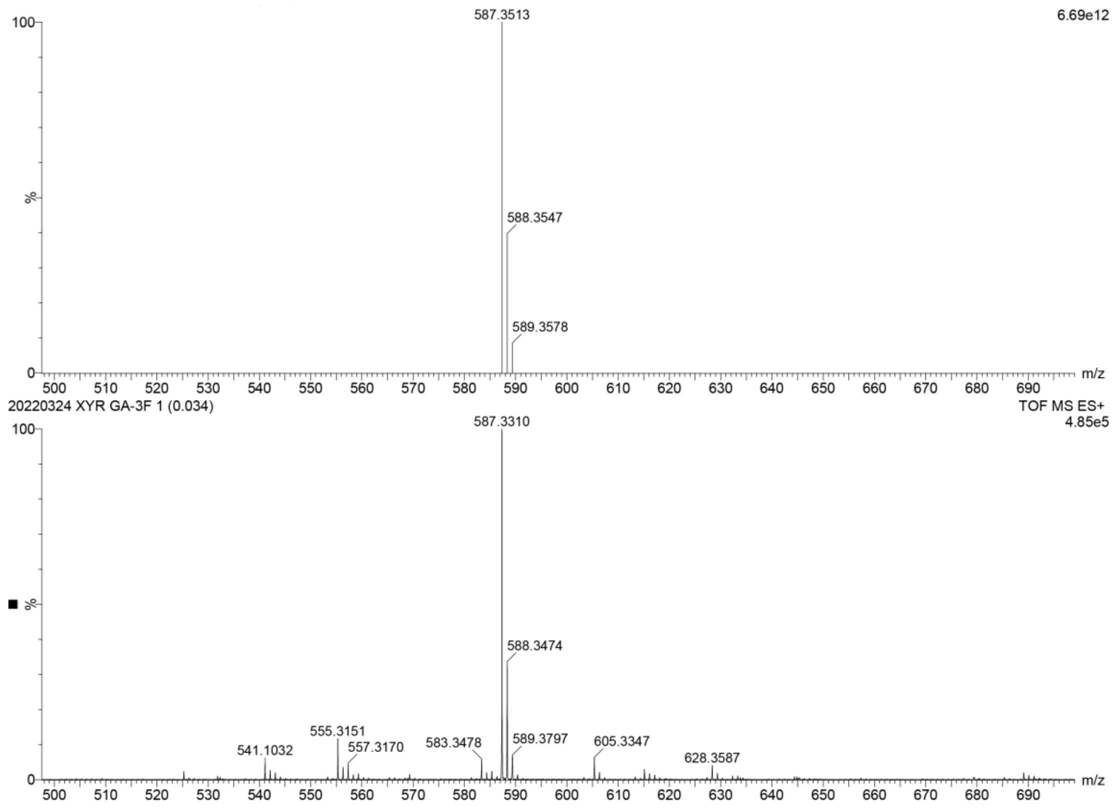

Figure S10 ^{1}H NMR spectra for **3d**

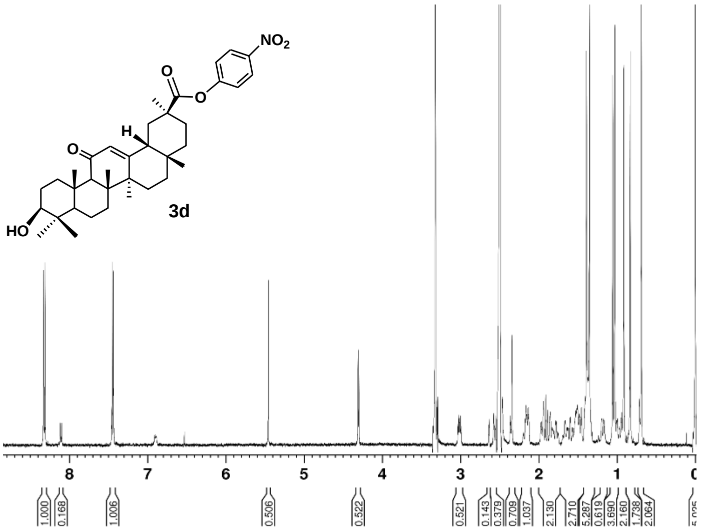

Figure S11 ^{13}C NMR spectra for **3d**

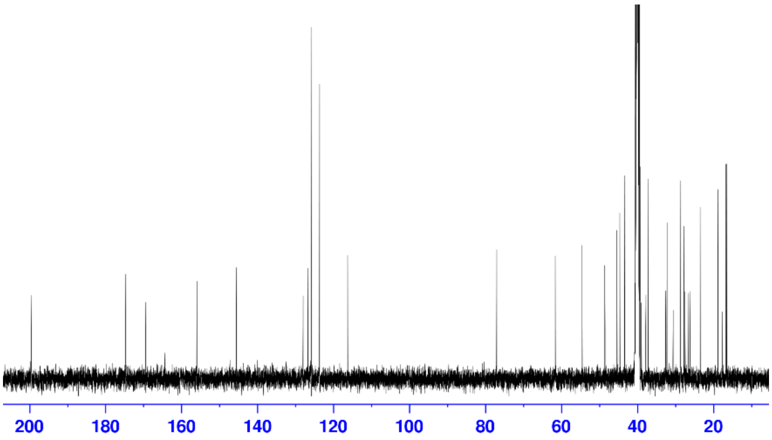

Figure S12 HRMS spectra for **3d**

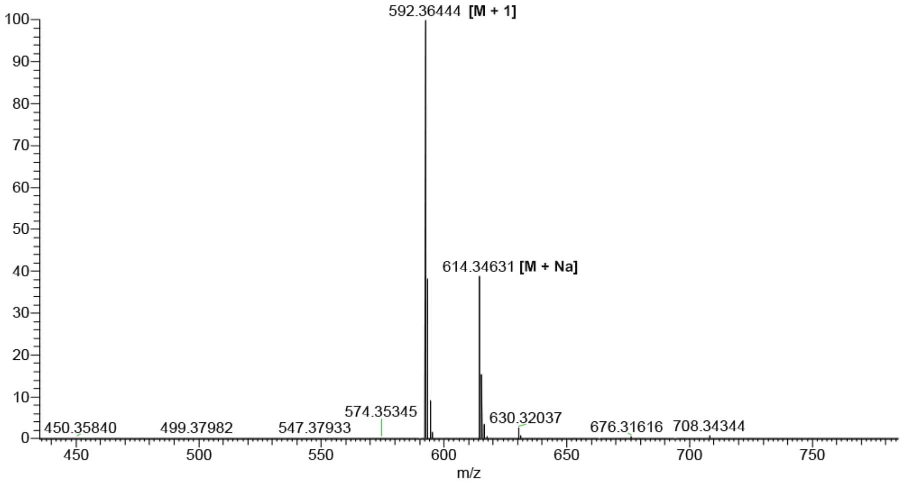

Figure S13 ¹H NMR spectra for **3e**

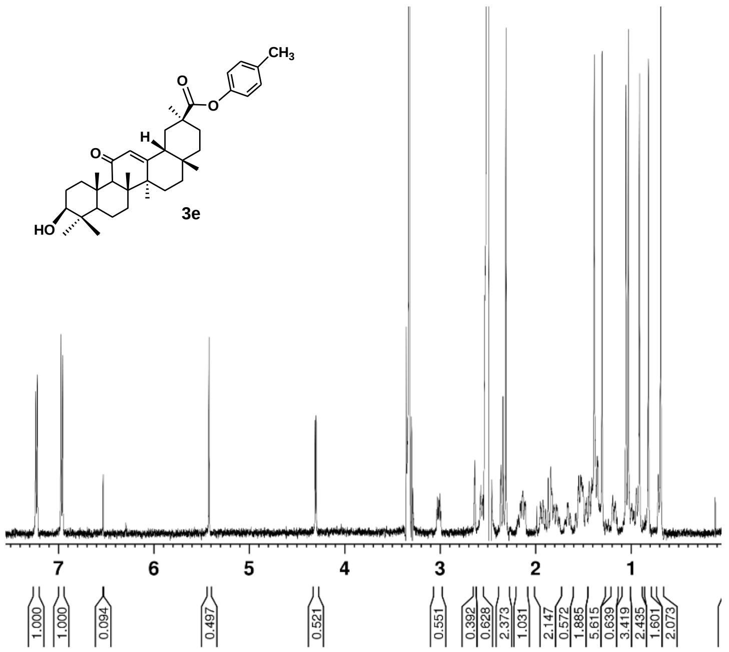

Figure S14 ¹³C NMR spectra for **3e**

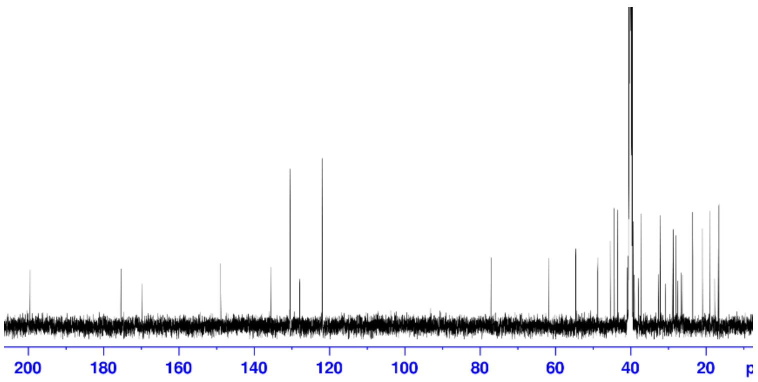

Figure S15 HRMS spectra for **3e**

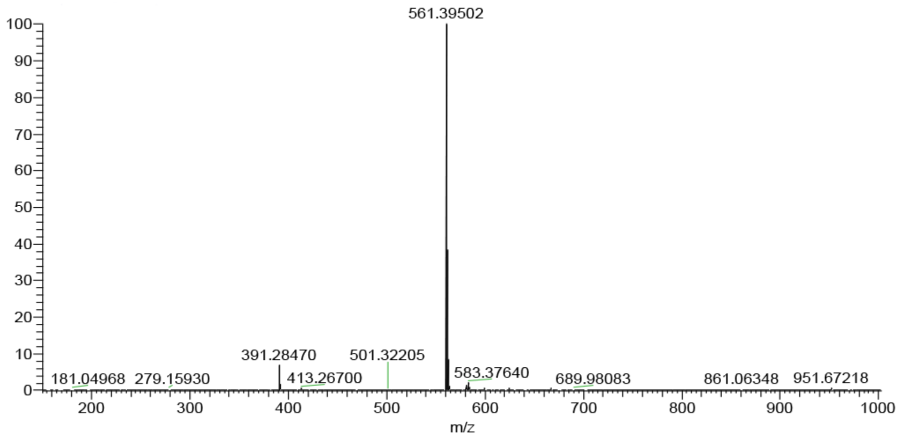

Figure S16 ^{1}H NMR spectra for **3f**

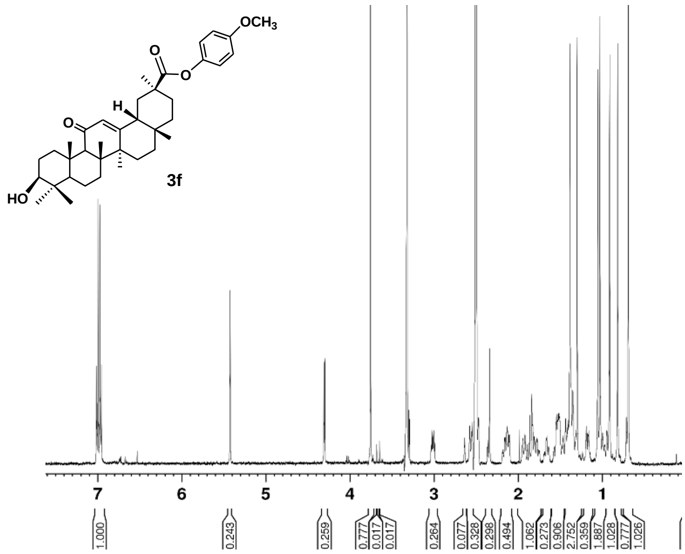

Figure S17 ^{13}C NMR spectra for **3f**

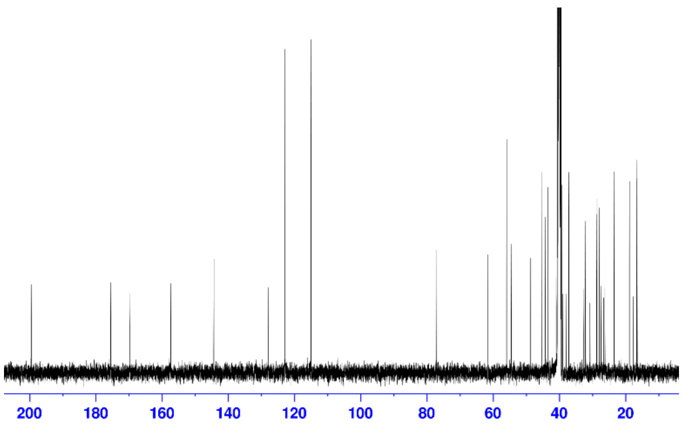

Figure S18 HRMS spectra for **3f**

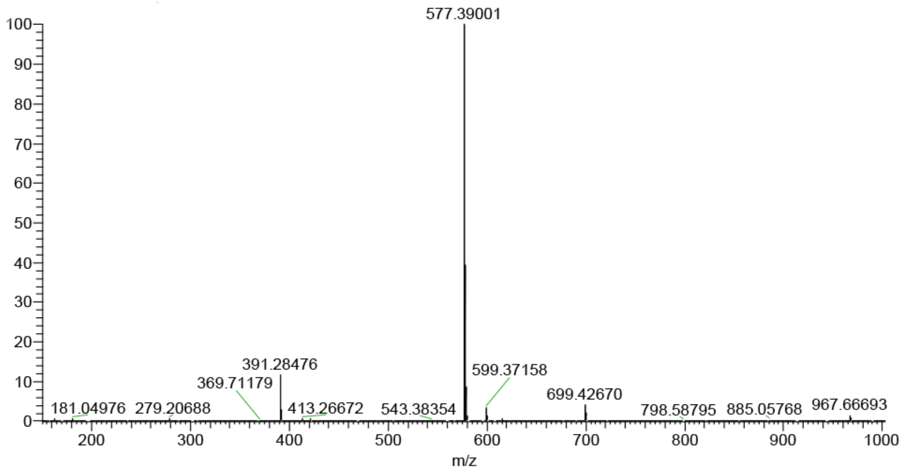

Figure S19 The purity of compound 3a was detected by HPLC

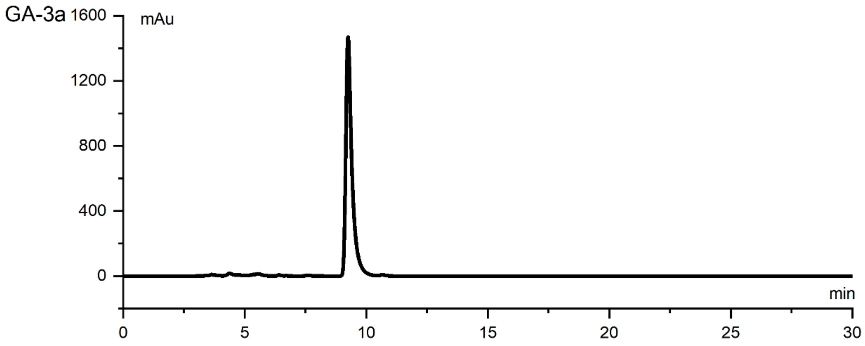

Supplement: Supplementary file 1 [file molecules-28-03164-s001.zip › molecules-2276608-supplementary.pdf]
